# Impact of Senolytic Treatment on Gene Expression in Aged Lung

**DOI:** 10.3390/ijms24087628

**Published:** 2023-04-21

**Authors:** Soo Jung Cho, Alexander Pronko, Jianjun Yang, Heather Stout-Delgado

**Affiliations:** Pulmonary and Critical Medicine, Department of Medicine, Weill Cornell Medicine, New York, NY 10065, USA

**Keywords:** senescence, lung, senolytics, chronic inflammation, aging

## Abstract

Cellular senescence plays a key role in mediating tissue remodeling and modulation of host responses to pathogenic stimuli. Our current study was designed to gain a better understanding of the impact of short-term senolytic treatment or inflammatory stimulation on lung senescence. The results of our study demonstrate that short term treatment of aged adult mice (20 months of age) with senolytics, quercetin, and dasatinib decreases p16 and p21 expression in lung tissue. Short-term treatment with senolytics also significantly improved the expression of genes associated with genomic instability, telomere attrition, mitochondrial dysfunction, DNA binding, and the inflammatory response. In contrast, in response to low-dose LPS administration, there was increased expression of genes associated with genomic instability, mitochondrial dysfunction, and heightened inflammatory responses in young adult murine lung (3 months of age). Taken together, the results of our current study illustrate the efficacy of senolytic treatment on modulating responses in aged lung and the potential role of chronic low dose inflammation on senescence induction in the lung.

## 1. Introduction

Aging Lung and Acute Respiratory Distress Syndrome (ARDS)*:* Age-associated changes in intrinsic mechanisms that aid in cell regeneration and repair contribute to an inability of lung cells to maintain baseline homeostasis. ARDS, the most severe form of acute lung injury (ALI), is a serious respiratory illness, with older patients having a higher risk for development [1]. Severe pneumonia, a common cause for ARDS development, is also a frequent complication of ARDS and can contribute to prolonged respiratory failure [1,2].

Role of Senescence in Lung Responses: In healthy individuals, immunosenescence begins around age 50 and contributes to impaired vaccine responsiveness, increased susceptibility to infection and autoimmunity, and chronic inflammation [3]. Cellular senescence plays a key role in mediating tissue remodeling and modulation of host responses to pathogenic stimuli. Previous work has demonstrated that reactive oxygen species (ROS), endoplasmic reticulum (ER) stress, epigenetic modifications, oncogene activation, mitochondrial dysfunction, and radiation-induced DNA damage can contribute to senescence development in the lung [4,5,6,7,8]. Senescence-associated secretory phenotype (SASP) is associated with cellular secretions of inflammatory mediators, including cytokines, matrix remodeling proteins, and other factors that contribute to the autocrine/paracrine cellular microenvironment of the lung [9]. A key feature of senescent cells is their longevity and resistance to apoptosis, collectively referred to as senescent cell anti-apoptotic pathways (SCAPs). Cellular senescence can interfere with wound healing in the lung. Inflammation-mediated cellular senescence can decrease fibroblast proliferation and migration [10]. Conversely, senescent lung fibroblasts can induce alveolar epithelial cell (AEC) cycle arrest, resulting in re-epithelialization [11,12,13]. Recent work has highlighted a role for the aged lung microenvironment in modulating dysfunctional baseline and post-influenza responses in alveolar macrophages (AM) [14]. Given the important role of tissue-resident AMs in host defense, catabolizing alveolar surfactant, and remodeling of injured tissue, age-associated alterations in AM function can have a deleterious impact on lung tissue homeostasis. Changes in AMs that occur with aging are not cell autonomous, are independent of circulating growth factors, and are directly modified by changes in the alveolar lining fluid [14].

Therapeutic Potential of Senolytic Interventions: Senescent cells, which occur in response to an accumulation of irreparable cellular damage occurring after stress or natural aging, can impair tissue function. Multiple senolytic drugs have been developed to overcome apoptosis resistance in senescent cells, and therapeutic interventions have the potential to delay features of aging. Quercetin, a natural flavonoid that binds to BCL-2, has been shown previously to eliminate senescent vascular smooth muscle cells and endothelial cells by inducing AMPK-mediated apoptosis, promoting mitophagy, and NRF2–NFkB signaling [15,16,17]. Recent work has demonstrated that quercetin treatment can also significantly reduce pneumolysin-induced hemolytic activity and cytotoxicity, inhibit choline kinase, and impair biofilm formation during bacterial infection [18,19,20]. Similarly, quercetin has been shown to exhibit antiviral properties and can inhibit influenza A entry and release [21,22,23,24]. Combined therapeutic intervention with quercetin and dasatinib (Q + D) can improve cellular function in aged hosts [25,26,27]. Treatment with Q + D has been shown to reduce senescent cell burden, improve physical performance, decrease proinflammatory cytokine secretion in human adipose tissue, improve survival during microbial exposure, and modulate the gut microbiome [27,28,29]. Importantly, preliminary clinical trial findings have demonstrated that Q + D treatment in humans, in addition to being safe and well tolerated, can significantly decrease senescent cell burden [30]. In addition, mice receiving two, one-week apart pre-treatments of Q + D had restored differentiation of CD4^+^ T helper cell populations in the lung during influenza [31]. Despite these advancements in our current knowledge about senolytics, very little is currently known regarding the impact of a sustained therapeutic regimen of quercetin, dasatinib, or Q + D on modulating molecular and cellular responses in aged lung.

Our current study was designed to gain a better understanding of the impact of short-term senolytic treatment or inflammatory stimulation on lung senescence. Results of our study demonstrate that short-term treatment of aged adult mice with senolytics, quercetin, and dasatinib decreases p16 and p21 expression in lung tissue. Short-term treatment with senolytics significantly decreased HMGB1 levels in plasma and was associated with improved expression of genes associated with genomic instability, telomere attrition, mitochondrial dysfunction, DNA binding, and the inflammatory response. In contrast, inflammation, in response to low-dose LPS administration, significantly enhanced HGMB1 levels in BAL and plasma of young adult mice. These levels were associated with increased expression of genes associated with genomic instability, mitochondrial dysfunction, and heightened inflammatory responses. Taken together, the results of this study illustrate the efficacy of senolytic treatment on modulating responses in aged lung and the potential role of chronic low dose inflammation on senescence induction in the lung.

## 2. Results

Our current study was designed to examine the impact of aging on senescence gene expression in the aged lung at baseline and in response to senolytic treatment. We also investigated the role of low-dose treatment of young mice with LPS, a pathogen associated molecular pattern (PAMP) that binds to TLR4, on inflammation-induced senescence gene expression in young lung.

### 2.1. Treatment of Aged Mice with Senolytics Reduced p16 and p21 Gene Expression in Lung

**Figure 1:**
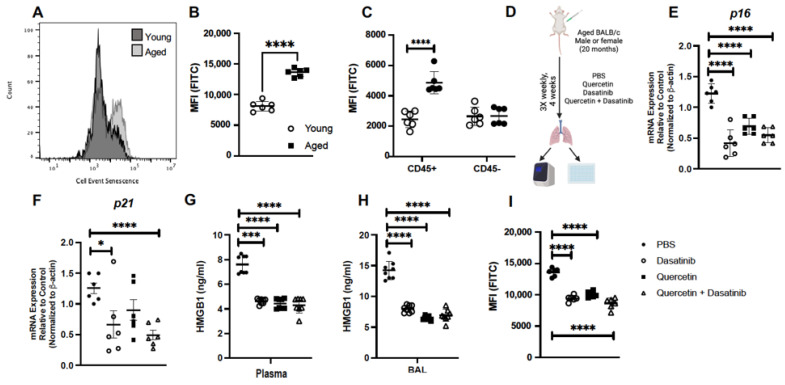
Treatment of Aged Mice with Senolytics Reduced *p16* and *p21* Gene Expression in Lung. (**A**–**C**) Baseline β-galactosidase hydryolysis in lung cells isolated from young (3 months) and aged (20 months) lung cells. (**C**) Lung cells were enriched for CD45, and β-galactosidase hydryolysis was assessed in CD45^+^ and CD45^−^ populations. (**D**) Experimental layout for senolytic treatment regimen in aged adult BALB/c mice (20 months). Lung tissue was collected from control and treated mice and expression of (**E**) *p16* and (**F**) *p21* was assessed by real-time PCR. (**G**) Plasma and (**H**) BAL was isolated and HMGB1 was assessed. (**I**) β-galactosidase hydryolysis in aged lung in response to senolytic treatment was assessed by flow cytometry. N = 6 mice per group, and the experiment was repeated 3 times. * *p* < 0.05, *** *p* < 0.001, **** *p* < 0.0001. Figure 1D was prepared using BioRender.

Our initial experiments were designed to examine the level of senescence present in aged lung at baseline. For these experiments, using total lung cells from gently dissociated lung, we investigated β-galactosidase hydryolysis by flow cytometry. When compared to young lung cells, we observed a significant increase in β-galactosidase hydryolysis in aged lung cells (Figure 1A,B). We next evaluated if differences in β-galactosidase hydryolysis were present in immune (CD45^+^) or non-immune (CD45^−^) cell populations. We observed a significant increase in β-galactosidase hydryolysis observed in aged CD45^+^ lung cells when compared to young (Figure 1C). Interestingly, we did not observe differences in β-galactosidase hydryolysis in young and aged unstimulated CD45^−^ lung cells (Figure 1C). Based on efficacy in previous studies, we chose to use senolytics quercetin (Q), dasatinib (D), and quercetin + dasatinib (Q + D) to investigate the impact of treatment on senescence gene expression in the aged lung. For these experiments, we treated aged (20 months) male and female BALB/c mice 3 times weekly for 4 weeks prior to isolation and analysis of lung tissue (Figure 1D). In response to the weekly treatment, there was a significant decrease in *p16* gene expression detected in response to Q, D, and Q + D when compared to PBS controls (Figure 1E). In response to Q + D, there was a significant decrease in *p21* gene expression quantified in aged lung (Figure 1F). We next evaluated if weekly treatment with senolytics might modulate the expression of damage-associated molecular pattern (DAMP) molecules, such as high mobility group box protein 1 (HMGB1). Plasma was isolated from PBS and senolytic treated mice and HMGB1 levels were assessed. When compared to PBS controls, weekly treatment with Q, D, and Q + D resulted in a decrease in HMGB1 detected in plasma and BAL (Figure 1G,H). We next investigated if senolytic treatment impacted senescence in aged lung. In response to weekly treatment, we observed a significant decrease in β-galactosidase hydryolysis occurred in senolytic-treated mice when compared to PBS controls (Figure 1I). 

**Figure 2:**
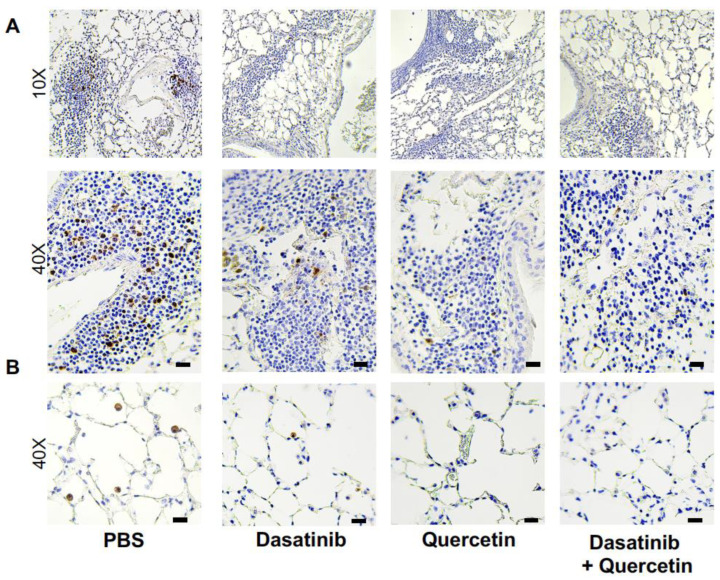
Treatment of Aged Mice with Senolytics Reduces p16 Expression in Lung. Lung tissue was collected from PBS-, quercetin-, dasatinib-, and quercetin + dasatinib-treated aged adult BALB/c mice (20 months of age). Immunohistochemical staining for p16 was performed on lung tissue sections and representative images were collected. (**A**) 10× and 40× images of lung lymphoid tissue and (**B**) alveolar space were observed. N = 6 mice per group. Bar represents 25 μM.

Based on these results, we next examined if there were changes in senolytic markers observed in unstimulated aged lung in response to PBS or senolytic treatment. As shown in Figure 2, we observed p16 expression in immune cells present in lymphoid tissue of the lung (2A) as well as alveolar macrophages present in the pulmonary alveoli (2B). In response to senolytic treatment, there was a marked decrease in p16-positive cells present in aged lung (Figure 2A,B). Taken together, in the absence of physiological stimuli, there was increased senescence in aged cells present in lymphoid tissue and immune cells present in pulmonary alveoli that was reduced in response to senolytic treatment.

### 2.2. Treatment of Aged Mice with Senolytics Alters Senescence Gene Expression in Aged Lung

**Figure 3:**
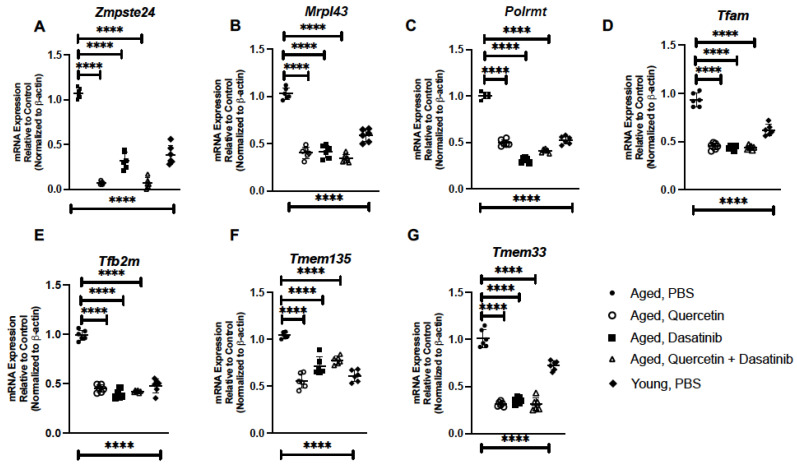
Treatment of Aged Mice with Senolytics Alters Senescence Gene Expression in Aged Lung. Lung tissue was collected from PBS-, quercetin-, dasatinib-, and quercetin + dasatinib-treated aged adult BALB/c mice (20 months of age). Expressions of (**A**) *Zmpste24*, (**B**) *Mrpl43*, (**C**) *Polrmt*, (**D**) *Tfam*, (**E**) *Tfb2m*, (**F**) *Tmem135*, and (**G**) *Tmem33* were assessed by real-time PCR. N = 6 mice per group, and the experiment was repeated 3 times. Young (3 months) PBS-treated lung expression analysis is included for comparison **** *p* < 0.0001.

We evaluated the impact of senolytic treatment on the expression of senescence genes in the aged lung. For these experiments, we isolated RNA from PBS and senolytic-treated lung and assessed gene expression by real-time PCR. In response to senolytics, there was a significant decrease in genes associated with genomic instability. Specifically, treatment with each of the senolytics resulted in decreased expression of zinc metalloprotease STE24 (*Zmpste24*) and mitochondrial ribosomal protein L43 (*Mrpl43*) (Figure 3A,B). Based on these findings, we investigated if senolytic treatment might contribute to changes in genes associated with maintaining mitochondrial replication. Transcription of mitochondrial DNA (mtDNA) is carried out by the mitochondrial RNA polymerase (POLRMT) and the mitochondrial transcription factors A (TFAM) and B2 (TF2BM) [32,33,34]. In response to Q, D, and Q + D treatment, there was a significant reduction in *Polrmt* expression that was associated with decreased *Tfam* and *Tfb2m* induction when compared to aged lung treated with PBS (Figure 3C–E). Transmembrane protein (TMEM) 135 has been previously shown to regulate mitochondrial dynamics, with a disruption in expression altering the fusion–fission balance of mitochondria [35]. Overexpression of TMEM135 can result in increased mitochondrial fragmentation and membrane potential whereas a loss of TMEM135 can decrease mitochondrial membrane potential and the rate of oxygen consumption [36]. In response to senolytic treatment, a significant decrease in *Tmem135* expression was observed in aged lung when compared to PBS controls (Figure 3F). Additional transmembrane proteins, such as TMEM33, have been shown to function as an ER stress-inducible transmembrane molecule that regulates expression of PERK and IRE1α [37]. Importantly, overexpression of TMEM33 can contribute to increased downstream ER stress signaling [37]. When compared to control, there was a significant reduction in *Tmem33* gene expression in aged lung in response to Q, D, and Q + D treatment (Figure 3G).

**Figure 4:**
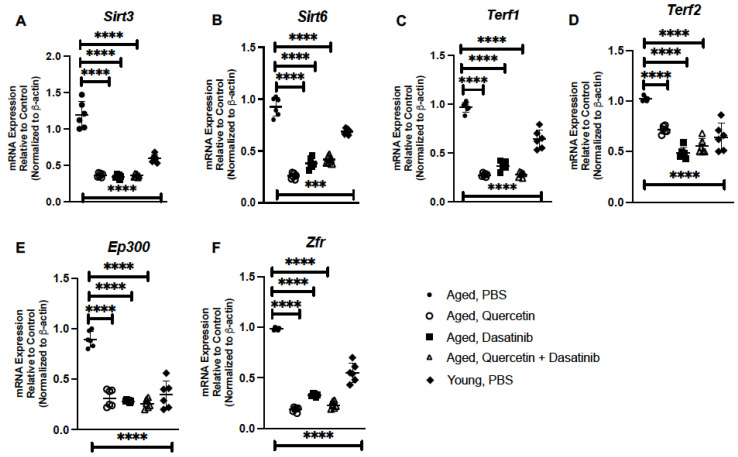
Treatment of Aged Mice with Senolytics Alters Senescence Modulatory Genes in Aged Lung. Lung tissue was collected from PBS-, quercetin-, dasatinib-, and quercetin + dasatinib-treated aged adult BALB/c mice (20 months of age). Expressions of (**A**) *Sirt3,* (**B**) *Sirt6*, (**C**) *Terf1,* (**D**) *Terf2*, (**E**) *Ep300*, and (**F**) *Zfr* were assessed by real-time PCR. N = 6 mice per group, and the experiment was repeated 3 times. Young (3 months) PBS-treated lung expression analysis is included for comparison *** *p* < 0.001 and **** *p* < 0.0001.

Human studies have demonstrated that increased levels of sirtuin 3 (Sirt3) have been associated with frailty in older persons while overexpression of sirtuin 6 (Sirt6) has been shown to inhibit the inflammatory senescence-associated secretory phenotype (SASP) [38,39]. When compared to aged control lung, there was a significant decrease in *Sirt3* and *Sirt6* expression observed in response to senolytic treatment (Figure 4A,B). We next examined the impact of senolytic treatment on genes associated with telomere attrition. Previous work has demonstrated that long-term overexpression of telomeric repeat binding factors (TERF) 1 and 2 can result in progressive telomere shortening [40]. When compared to PBS-treated lung, there was a decrease in *Terf1* and *Terf2* expression observed in aged lung in response to senolytics (Figure 4C,D). We next examined the potential contribution of senolytics on the expression of DNA binding and transcriptional regulators, such as E1A-binding protein p300 (EP300) and zinc finger RNA-binding protein (ZFR), in aged lung. Previous work has demonstrated a slight increase in EP300 histone acetyltransferase activity in young and aged murine lung [41]. In response to Q, D, and Q + D treatment, we observed a significant decrease in *Ep300* expression (Figure 4E). Similarly, the expression of *Zfr* was also decreased in aged lung in response to senolytic treatment (Figure 4F).

### 2.3. Senolytic Treatment of Aged Mice Alters Inflammatory Gene Expression in Aged Lung

**Figure 5:**
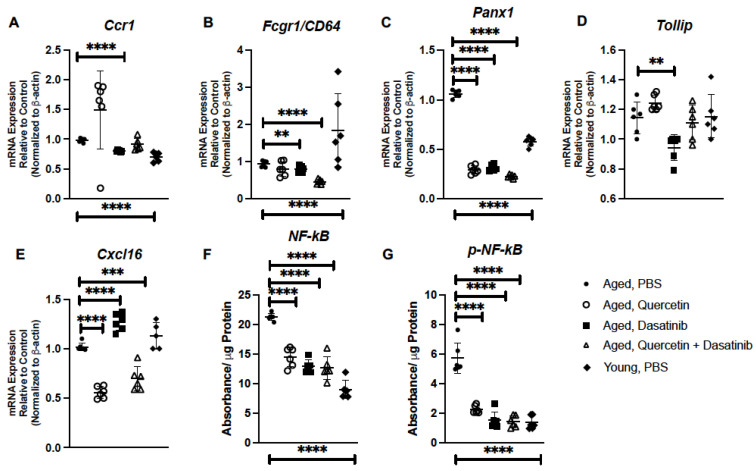
Senolytic Treatment of Aged Mice Alters Inflammatory Gene Expression in Aged Lung. Lung tissue was collected from PBS-, quercetin-, dasatinib-, and quercetin + dasatinib-treated aged adult BALB/c mice (20 months of age). Expressions of (**A**) *Ccr1,* (**B**) *Fcgr1/CD64*, (**C**) *Panx1,* (**D**) *Tollip*, and (**E**) *Cxcl16* were assessed by real-time PCR. Protein expression of (**F**) NF-κB and (**G**) p-NF-κB in aged lung was assessed by PathScan ELISA. N = 6 mice per group, and the experiment was repeated 3 times. Young (3 months) PBS-treated lung expression analysis is included for comparison. ** *p* < 0.01, *** *p* < 0.001, and **** *p* < 0.0001.

We evaluated if senolytic treatment of aged mice might contribute to changes in inflammatory gene expression in aged lung. Previous work has demonstrated age-associated changes in chemokine receptor CCR1 expression [42]. As CCR1 is critical for neutrophil recruitment, we quantified *Ccr1* gene expression in PBS- and senolytic-treated aged lung [43]. While similar expression of *Ccr1* was observed in aged lung isolated from PBS-, Q-, and Q + D-treated mice, there was significant decrease detected in lung of D-treated mice (Figure 5A). C-reactive protein binds to the receptor CD64 to induce NF-κB-mediated pro-inflammatory signaling, with excessive signaling potentially being a pathogenic mechanism that contributes to senescence [44]. In response to treatment with Q, D, and Q + D, we observed a significant decrease in *Cd64* expression in aged lung (Figure 5B). Pannexins (PANXs), such as PANX1, play a role in paracrine and autocrine immune cell communication [45,46]. Specifically, PANX1 channels are positive regulators of immune cell migration in response to different mechanical and chemical signals [47]. Given the role of PANX1 in cellular communication, we next evaluated the impact of senolytic treatment on *Panx1* expression in aged lung. In response to Q, D, and Q + D treatment, there was a significant decrease in *Panx1* expression detected in aged lung (Figure 5C). As toll interacting protein (TOLLIP) has been previously shown to play an important role in replicative senescence, we next investigated the impact of senolytics on *Tollip* expression in aged lung [48]. While there was similar expression in lung isolated from PBS-, Q-, and Q + D-treated mice, we observed a significant decrease in *Tollip* expression in lung isolated mice receiving an administration of D (Figure 5D). CXCL16 is a chemokine with structural similarity to fractalkine that expresses on the surface of antigen-presenting cells, including subsets of CD19^+^ B cells and CD14^+^ monocytes/macrophages [49]. In response to Q or Q + D treatment, there was a significant decrease in *Cxcl16* gene expression detected in aged lung (Figure 5E). Interestingly, in response to D treatment, there was a significant increase in *Cxcl16* expression when compared to aged control lung tissue (Figure 5E). We next investigated if treatment with senolytic compounds impacted NF-κB and p-NF-κB protein expression. When compared to PBS-treated controls, there was a significant decrease in NF-κB and p-NF-κB expression detected in aged lung in response to senolytic treatments (Figure 5F,G).

### 2.4. Treatment of Young Mice with Low-Dose LPS Induces HMGB1 Production

**Figure 6:**
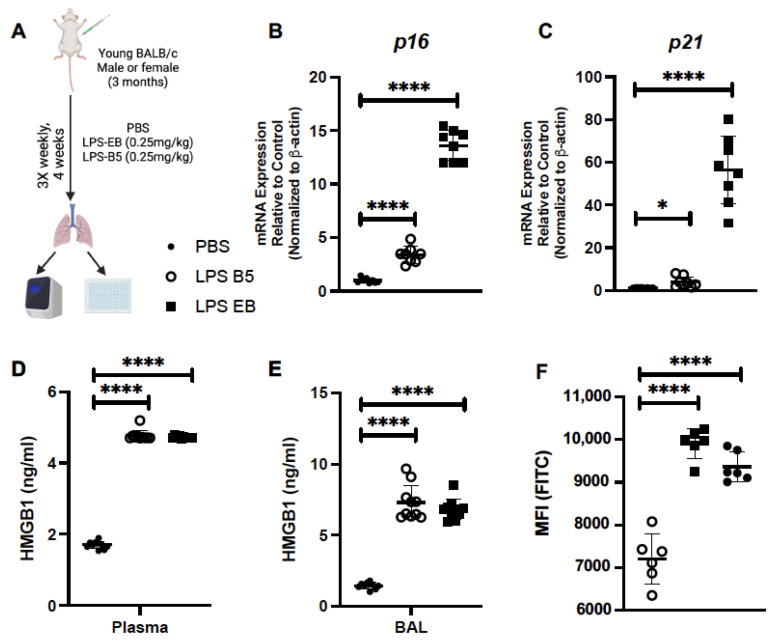
Treatment of Young Mice with Low-Dose LPS Induces HMGB1 Production. (**A**) Experimental layout for LPS injections in young adult BALB/c mice (3 months). Lung tissue was collected from control and treated mice. (**B**,**C**) *p16* and *p21* expression in young lung in response to low-dose LPS administration. (**D**) Plasma and (**E**) BAL was isolated and HMGB1 was assessed. (**F**) β-galactosidase hydryolysis in young lung in response to senolytic treatment was assessed by flow cytometry. N = 6 mice per group, and the experiment was repeated 3 times. * *p* < 0.05, **** *p* < 0.0001. Figure 6A was created using BioRender.

We next evaluated the impact of low-dose ‘sterile’ inflammatory stimuli, such as LPS, on the induction of lung senescence. For these experiments, young (3 months of age) mice were intraperitoneally injected with a low dose of LPS (3 times weekly for 4 weeks) (Figure 6A). We first investigated p16 and p21 mRNA expression in young lung in response to LPS. In response to repeated low-dose administration of LPS, there was a significant increase in *p16* (Figure 6B) and *p21* (Figure 6C). In response to LPS, there was a significant elevation in HMGB1 detected in plasma (Figure 6D) and BAL (Figure 6E) when compared to mice receiving PBS alone. We next investigated if low-dose LPS impacted senescence in young lung. In response to weekly treatment, we observed that a significant increase in β-galactosidase hydryolysis occurred in young, LPS-treated mice when compared to PBS controls (Figure 6F).

### 2.5. Treatment of Young Mice with Low-Dose LPS Alters Senescence Gene Expression in Lung

**Figure 7:**
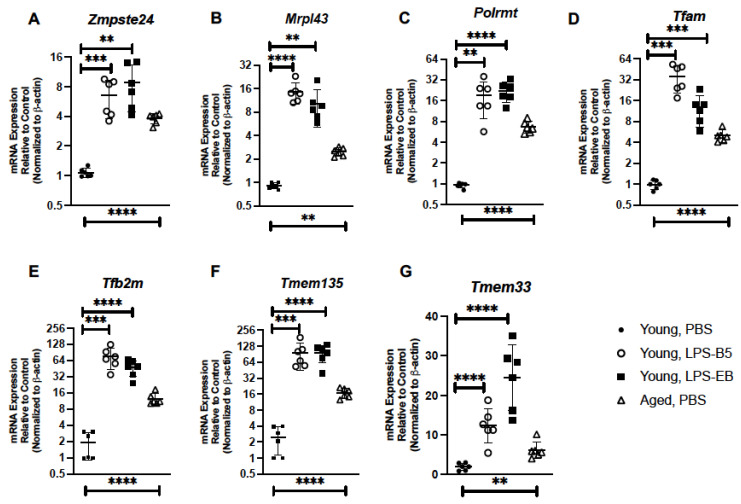
Treatment of Young Mice with Low-Dose LPS Alters Senescence Gene Expression in Lung. Lung tissue was collected from PBS-, LPS-B5-, and LPS-EB-treated young adult BALB/c mice (3 months of age). Expressions of (**A**) *Zmpste24*, (**B**) *Mrpl43*, (**C**) *Polrmt*, (**D**) *Tfam*, (**E**) *Tf2m*, (**F**) *Tmem135*, and (**G**) *Tmem33* were assessed by real-time PCR. Aged (20 months) PBS-treated lung expression analysis is included for comparison. N = 6 mice per group, and the experiment was repeated 3 times. ** *p* < 0.01, *** *p* < 0.001, and **** *p* < 0.0001.

Based on these findings, we examined if low-dose administration of LPS might alter senescence gene expression in the young lung. For these experiments, we isolated RNA from lung tissue collected from young mice receiving PBS, ultrapure LPS-B5, or ultrapure LPS-EB and examined gene expression by real-time PCR. In response to administration of LPS-B5 or LPS-EB, there was a significant elevation in *Zmpste24* and *Mrpl43* expression in lung when compared to PBS controls (Figure 7A,B). We next evaluated if low-dose LPS treatment might contribute to changes in genes associated with maintaining mitochondrial replication. When compared to control, there was a significant elevation of *Polrmt, Tfam*, and *Tfb2m* in lung tissue collected from LPS-treated mice (Figure 7C–E). As TMEM135 and TMEM33 play important roles in maintaining mitochondrial integrity and induction of ER stress responses, respectively, we examined if low-dose LPS administration might impact expression in young lung. In response to LPS-B5 or LPS-EB, elevated expression of *Tmem135* and *Tmem33* was observed in young lung when compared to PBS controls (Figure 7F,G).

**Figure 8:**
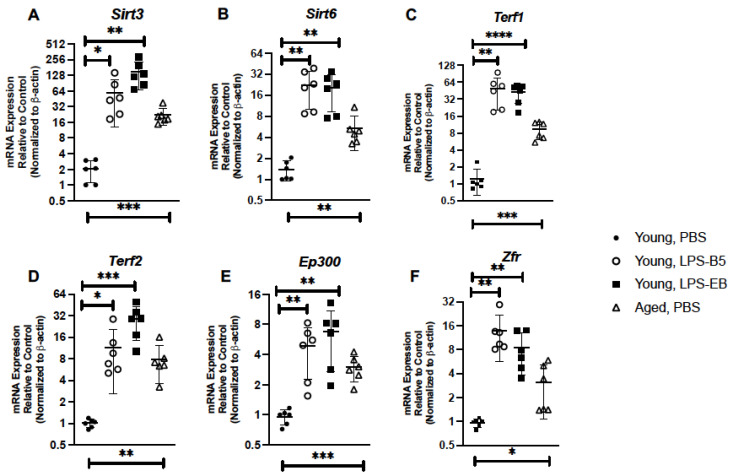
Treatment of Young Mice with Low-Dose LPS Alters Senescence Modulatory Genes in Lung. Lung tissue was collected from PBS-, LPS-B5-, and LPS-EB-treated young adult BALB/c mice (3 months of age). Expressions of (**A**) *Sirt3*, (**B**) *Sirt6*, (**C**) *Terf1*, (**D**) *Terf2*, (**E**) *Ep300*, and (**F**) *Zfr* were assessed by real-time PCR. Aged (20 months) PBS-treated lung expression analysis is included for comparison. N = 6 mice per group, and the experiment was repeated 3 times. * *p* < 0.05, ** *p* < 0.01, *** *p* < 0.001, and **** *p* < 0.0001.

Given the importance of sirtuins 3 and 6 in modulating senescence, we evaluated if the expressions of these genes were altered in young lung in response to LPS administration. When compared to PBS controls, there was a significant increase in both *Sirt3* and *Sirt6* expression detected in lung collected from LPS-treated mice (Figure 8A,B). When compared to PBS-treated lung, there was an increase in *Terf1* and *Terf2* expression observed in young lung in response to LPS treatment (Figure 8C,D).We next examined the impact that low-dose, repeated LPS administration might have on DNA binding proteins, EP300, and ZFR. There was a significant increase in *Ep300* and *Zfr* gene expression in young lung in response to LPS when compared to PBS controls (Figure 8E,F).

### 2.6. Treatment of Young Mice with Low-Dose LPS Alters Inflammatory Gene Expression in Young Lung

**Figure 9:**
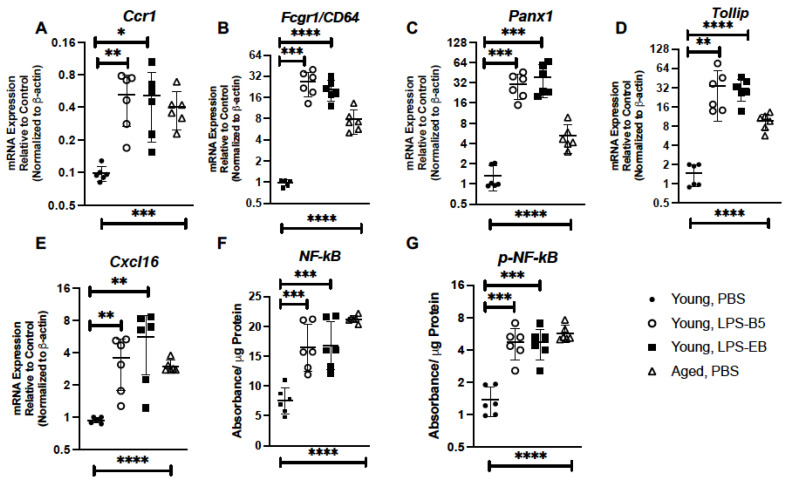
Treatment of Young Mice with Low-Dose LPS Alters Inflammatory Gene Expression in Lung. Lung tissue was collected from PBS-, LPS-B5-, and LPS-EB-treated young adult BALB/c mice (3 months of age). Expressions of (**A**) *Ccr1*, (**B**) *Fcgr1/CD64*, (**C**) *Panx1*, (**D**) *Tollip*, and (**E**) *Cxcl16*, were assessed by real-time PCR. Protein expression of (**F**) NF-κB and (**G**) p-NF-κB in young lung was assessed by PathScan ELISA. Aged (20 months) PBS-treated lung expression analysis is included for comparison. N = 6 mice per group, and the experiment was repeated 3 times. * *p* < 0.05, ** *p* < 0.01, *** *p*< 0.001, and **** *p* < 0.0001.

We next evaluated if repeated treatment with low-dose LPS could impact inflammatory gene expression in the young lung. In response to LPS, there was a significant elevation in *Ccr1* expression in young lung when compared to PBS controls (Figure 9A). Given the importance of CD64, PANX1, TOLLIP, and CXCL16 to the immune response, we examined if the expressions of these genes were altered in young lung isolated from LPS-treated mice. When compared to PBS controls, there was a significant elevation of Cd64, Panx1, Tollip, and Cxcl16 detected in young lung in response to LPS-B5 and LPS-EB (Figure 9B–E). Based on these findings, we next investigated the impact of LPS-B5 and LPS-EB treatment on NF-κB and p-NF-κB protein expression in young lung. When compared to PBS-treated controls, there was a significant difference in NF-κB and p-NF-κB detected in young lung in response to LPS treatment (Figure 9F,G).

## 3. Discussion

Our current study was designed to gain a better understanding of the impact of short-term senolytic treatment or inflammatory stimulation on lung senescence. The results of our study demonstrate that short-term treatment of aged adult mice with senolytics, quercetin (Q) and dasatinib (D) significantly decreased HMGB1 levels in plasma and was associated with improved expression of gene associated with genomic instability, telomere attrition, mitochondrial dysfunction, DNA binding, and the inflammatory response. In contrast, inflammation, in response to low-dose LPS administration, significantly enhanced HGMB1 levels in BAL and plasma of young adult mice. These levels were associated with increased expression of genes associated with genomic instability, mitochondrial dysfunction, and heightened inflammatory responses.

Previous work has demonstrated that in the absence of ZMPSTE24, there is accelerated aging [50]. Importantly, in the absence of ZMPSTE24, there was a significant reduction in the proliferation of fibroblasts, with many exhibiting enhanced senescence-associated β-galactosidase levels when compared to age-matched controls [50]. In our study, we observed an increase in *Zmpste24* gene expression occurred in young lung in response to low-dose inflammatory stimulation. Elevated *Zmpste24* expression may serve as a protective mechanism to prevent DNA damage and double-stranded breaks [51]. In this context, elevated *Zmpste24* may also be increased at baseline in aged lung as a feedback mechanism to maintain genomic integrity in the lung. Senolytic treatment, which has been shown to induce apoptosis of senescent cells, may help to restore homeostasis within aged lung tissue, improve genomic integrity, and result in decreased *Zmpste24* expression. It is important to note that *Zmpste24* expression was still detectable in all aged lung tissue, and mice did not exhibit any deleterious effects or lung tissue injury due to the senolytic treatment.

TMEM135 has been shown to localize on mitochondria, and mutations in TMEM135 can contribute to imbalanced mitochondrial dynamics [35]. Overexpression of TMEM135 can result in increased mitochondrial fragmentation and membrane potential whereas a loss of TMEM135 can decrease mitochondrial membrane potential and the rate of oxygen consumption [36]. Our findings demonstrate that, in response to senolytic treatment, there was a significant decrease in *Tmem135* expression in aged lung. It is possible that senolytic treatment and removal of senescent cells contributes to decreased oxidative stress and reduction of multiple genes associated with maintaining mitochondrial integrity. Importantly, *Tmem135* expression was still detectable in aged lung in response to senolytic treatment. As TMEM135 is critical for protection against environmental stress, elevated expression in young lung in response to LPS stimulation might be attributed to increased cellular stress occurring in lung in response to chronic low-dose inflammation.

Previous work has demonstrated that p16 and senescence-associated β-galactosidase can be induced in macrophages as part of a reversible response to physiological stimuli [52]. Importantly, these studies demonstrated that in response to poly(I:C) stimulation, there was no significant change in luciferase activity observed in lung isolated from 83-week-old p16^Ink4a/Luc^ mice [52]. It is plausible that peritoneal macrophages, as utilized in these studies, may be significantly influenced by the surrounding adipose tissue and upregulate senescence markers, such as p16 and β-galactosidase, in direct response to intraperitoneal inflammatory stimuli [52]. As the aged lung environment determines the unique phenotype of alveolar macrophages, it is possible that expression of these markers is dependent on environmental cues and is regulated differently in each organ system [53].

It is important to note several limitations of our current study. We examined gene expression in whole lung tissue samples, which contain a heterogeneous population of lung cells. The direct impact of senolytic treatment on specific cell subsets will need to be evaluated to determine if senolytic therapies directly impact one cell population, resulting in changes in senescence gene expression or in multiple cell populations within the lung. Conversely, the impact of low-dose LPS stimulation on senescent gene expression in specific cell populations will also need to be investigated in future studies. It is unclear if senescence gene expression in young lung is altered after LPS stimulation is no longer present. It will be important to examine changes in gene expression at later time points to determine if these values return to baseline in the young lung. Future work will need to be performed to evaluate the impact of senolytic treatment on low-dose LPS-mediated induction of senescence.

Another limitation of our current study is that we used LPS as a sterile inflammatory compound to induce inflammation in young lung. LPS-B5 and LPS-EB are highly pyrogenic prototypical endotoxins and potent activators of TLR4. While a low dose of LPS was used, it is important to note that repeated administration may produce an accelerated senescence phenotype that is not stereotypical of chronological aging. It is also possible that exposure to a suboptimal dose of LPS rendered immune cells, such as macrophages, tolerant to subsequent LPS exposures [54]. Thereby, heightened expression of senescence genes in young lung may reflect altered immune responses that correspond with the length of LPS duration.

Taken together, results of our current study illustrate the efficacy of senolytic treatment on modulating responses in aged lung and the potential role of chronic low-dose inflammation on senescence induction in the lung.

## 4. Materials and Methods

### 4.1. Mice

Male and female wild-type young (3 month, Charles Rivers Laboratories) and aged (18+ months) (NIA Rodent Colony, Charles Rivers Laboratories) BALB/c mice were housed in the WCM animal facility and handled under identical husbandry conditions and fed certified commercial feed. The IACUC at Weill Cornell Medicine approved the use of animals in this study, and methods were carried out in accordance with the relevant guidelines and regulations. No animals were used in the study if there was evidence of skin lesions, weight loss, or lymphadenopathy.

### 4.2. Senolytic Preparation and In Vivo Administration

Dasatinib and quercetin were dissolved in vehicle containing 10% ethanol, 30% polyethylene glycol 400, and 60% Phosal 50 PG at a concentration of 2 mg/mL and 20 mg/mL, respectively. Fresh solutions were prepared on the day of injection. Mice were injected intraperitoneally every other day (3 times per week) for 4 weeks (12 injections total) with PBS + vehicle, quercetin (0.625 mg/mouse), dasatinib (5 mg/kg), or quercetin (0.3125 mg/mouse) + dasatinib (2.5 mg/kg). To prevent inflammation, the injection site was alternated between administrations. Mouse weights and clinical scores were assessed daily for the duration of treatment. No adverse side effects or weight loss were observed in treated mice.

### 4.3. LPS Preparation and In Vivo Administration

Ultrapure LPS-EB (catalog #: tlrl-3pelps) and LPS-B5 (catalog #: tlrl-pb5lps) were purchased from Invivogen. Compounds were resuspended per the manufacturers’ instruction, and aliquots were stored at −20 °C until preparation for injection. Fresh dilutions of each LPS compound were made on the day of injection. Mice were injected intraperitoneally with PBS, LPS-EB (0.25 mg/kg), or LPS-B5 (0.25 mg/kg). To prevent inflammation, the injection site was alternated between administrations. Mouse weights and clinical scores were assessed daily for the duration of treatment. No adverse side effects or weight loss were observed in treated mice.

### 4.4. RNA Purification and Real-Time PCR

RNA samples were extracted from lung tissue using the automated Maxwell RNA extraction protocol (Madison, WI, USA). Samples were quantified and A_260/280_ ratios were recorded. Samples were reverse transcribed using the First Stand Synthesis Kit and quantified using the RT^2^ Profiler^TM^ PCR Assays (Qiagen: Mouse Senescence, PAMM-178Z, Hilden, Germany). PCR efficiency and reproducibility were assessed, and results were quantified using analytical software provided by Qiagen Gene Globe. Comparison of housekeeping gene expression is shown in Supplemental Figure S1.

### 4.5. HMGB1 Assay

Blood and bronchoalveolar lavage (BAL) were collected from euthanized mice. Plasma was isolated after centrifugation (7000 rpm, 10 min) and BAL was clarified (1200 rpm, 10 min) (ThermoFisher Scientific, Waltham, MA, USA). HMGB1 levels were assessed using the HMGB1 kit (Perkin Elmer/CisBio, catalog #: 62HMGPEG) per manufacturer’s instructions.

### 4.6. CellEvent Senescence Green Detection Assay

Whole lung cell populations were isolated from fresh lung tissue. Briefly, lung tissue was dissociated per the manufacturer’s instructions using the Lung Dissociation kit (Miltenyi Biotec). Whole lung cell populations or CD45^+^ and CD45^−^ enriched populations (Miltenyi Biotec using anti-CD45 microbeads) were counted, and 1 × 10^6^ cells per tube were treated with 1:1000 dilution of CellEvent Senescence probe, pH 6.0 (ThermoFisher Scientific, Catalog #: C10840) and incubation at 37 °C for 2 h. Samples were washed with PBS (+1% BSA). Fluorescence was quantified using the BD Accuri c6 cytometer and analyzed by FlowJo Software, version 10.

### 4.7. PathScan ELISA

Homogenates were prepared from whole lung cell populations (1 × 10^6^ cells/mL) using 1× Cell Lysis Buffer containing protease inhibitors (Cell Signal Technology, Catalog #: 9803) per manufacturer’s instructions. NF-κB (Cell Signal Technology, Catalog #: 7174C) and p-NF-κB (Cell Signal Technology, Catalog #: 7173C) were quantified and normalized to protein concentration.

### 4.8. p16 Immunohistochemistry of Lung Tissue

Mice were euthanized, and right lung tissue was collected for downstream analysis. To maintain architecture, left lung was distended with 1% low-melting agarose and placed into cold formalin [55]. Tissue samples were processed by the Translational Research Program at WCM Pathology and Laboratory of Medicine. Immunohistochemistry was performed per the antibody manufacturer’s recommendation (Abcam, Cambridge, UK, AB211542, anti-CDKN2A/p16INK4a). Tissue was treated with a 1:250 dilution of anti-p16INK4a in SignalStain antibody diluent (Cell Signal Technology, catalog #8112) overnight at 4 °C prior to treatment with secondary SignalStain Boost IHC detection reagent (Cell Signal Technology, catalog#8114) and DAB SignalStain substrate (Cell Signal Technology, catalog #8059). Counterstaining with hematoxylin was performed. Images were scanned using the EVOS FL Auto Imaging System (ThermoFisher Scientific, Waltham, MA, USA).

### 4.9. Statistical Analysis

Comparison of groups was performed using a two-tailed t-test, and comparisons between groups were verified by one-way ANOVA. For two component comparisons, two-way ANOVA was used to calculate statistical significance. All samples were independent and contained the same sample size for analysis. All data were analyzed using GraphPad Prism software. Statistical significance was considered by a * *p* < 0.05, ** *p* < 0.01, *** *p* < 0.001, and **** *p* < 0.0001.

## Data Availability

Data presented in this study is provided within the article.

## Supplementary Materials

The supporting information can be downloaded at: https://www.mdpi.com/article/10.3390/ijms24087628/s1.

## References

[1] Ely E.W., Wheeler A.P., Thompson B.T., Ancukiewicz M., Steinberg K.P., Bernard G.R. (2002). Recovery rate and prognosis in older persons who develop acute lung injury and the acute respiratory distress syndrome. Ann. Intern. Med..

[2] Siner J.M., Pisani M.A. (2007). Mechanical ventilation and acute respiratory distress syndrome in older patients. Clin. Chest Med..

[3] Rubelt F., Sievert V., Knaust F., Diener C., Lim T.S., Skriner K., Klipp E., Reinhardt R., Lehrach H., Konthur Z. (2012). Onset of immune senescence defined by unbiased pyrosequencing of human immunoglobulin mRNA repertoires. PLoS ONE.

[4] Bikkavilli R.K., Avasarala S., Van Scoyk M., Arcaroli J., Brzezinski C., Zhang W., Edwards M.G., Rathinam M.K., Zhou T., Tauler J. (2015). Wnt7a is a novel inducer of beta-catenin-independent tumor-suppressive cellular senescence in lung cancer. Oncogene.

[5] Childs B.G., Durik M., Baker D.J., van Deursen J.M. (2015). Cellular senescence in aging and age-related disease: From mechanisms to therapy. Nat. Med..

[6] Citrin D.E., Shankavaram U., Horton J.A., Shield W., Zhao S., Asano H., White A., Sowers A., Thetford A., Chung E.J. (2013). Role of type II pneumocyte senescence in radiation-induced lung fibrosis. J. Natl. Cancer Inst..

[7] Liu J., Yang J.R., Chen X.M., Cai G.Y., Lin L.R., He Y.N. (2015). Impact of ER stress-regulated ATF4/p16 signaling on the premature senescence of renal tubular epithelial cells in diabetic nephropathy. Am. J. Physiol. Cell Physiol..

[8] Sidler C., Kovalchuk O., Kovalchuk I. (2017). Epigenetic Regulation of Cellular Senescence and Aging. Front Genet..

[9] Ozcan S., Alessio N., Acar M.B., Mert E., Omerli F., Peluso G., Galderisi U. (2016). Unbiased analysis of senescence associated secretory phenotype (SASP) to identify common components following different genotoxic stresses. Aging.

[10] Bang M., Ryu O., Kim D.G., Mabunga D.F., Cho K.S., Kim Y., Han S.H., Kwon K.J., Shin C.Y. (2019). Tenovin-1 Induces Senescence and Decreases Wound-Healing Activity in Cultured Rat Primary Astrocytes. Biomol. Ther..

[11] Blokland K.E.C., Pouwels S.D., Schuliga M., Knight D.A., Burgess J.K. (2020). Regulation of cellular senescence by extracellular matrix during chronic fibrotic diseases. Clin. Sci..

[12] Blokland K.E.C., Waters D.W., Schuliga M., Read J., Pouwels S.D., Grainge C.L., Jaffar J., Westall G., Mutsaers S.E., Prele C.M. (2020). Senescence of IPF Lung Fibroblasts Disrupt Alveolar Epithelial Cell Proliferation and Promote Migration in Wound Healing. Pharmaceutics.

[13] Schuliga M., Read J., Blokland K.E.C., Waters D.W., Burgess J., Prele C., Mutsaers S.E., Jaffar J., Westall G., Reid A. (2020). Self DNA perpetuates IPF lung fibroblast senescence in a cGAS-dependent manner. Clin. Sci..

[14] McQuattie-Pimentel A.C., Ren Z., Joshi N., Watanabe S., Stoeger T., Chi M., Lu Z., Sichizya L., Aillon R.P., Chen C.I. (2021). The lung microenvironment shapes a dysfunctional response of alveolar macrophages in aging. J. Clin. Investig..

[15] Jiang Y.H., Jiang L.Y., Wang Y.C., Ma D.F., Li X. (2020). Quercetin Attenuates Atherosclerosis via Modulating Oxidized LDL-Induced Endothelial Cellular Senescence. Front. Pharmacol..

[16] Shao Y., Sun L., Yang G., Wang W., Liu X., Du T., Chen F., Jing X., Cui X. (2022). Icariin protects vertebral endplate chondrocytes against apoptosis and degeneration via activating Nrf-2/HO-1 pathway. Front. Pharmacol..

[17] Wang L., Ma Y., Wei W., Wan P., Liu K., Xu M., Cong S., Wang J., Xu D., Xiao Y. (2020). Cadherin repeat 5 mutation associated with Bt resistance in a field-derived strain of pink bollworm. Sci. Rep..

[18] Lv Q., Zhang P., Quan P., Cui M., Liu T., Yin Y., Chi G. (2020). Quercetin, a pneumolysin inhibitor, protects mice against Streptococcus pneumoniae infection. Microb. Pathog..

[19] Wang J., Song M., Pan J., Shen X., Liu W., Zhang X., Li H., Deng X. (2018). Quercetin impairs Streptococcus pneumoniae biofilm formation by inhibiting sortase A activity. J. Cell Mol. Med..

[20] Zimmerman T., Ibrahim S.A. (2022). Quercetin Is a Novel Inhibitor of the Choline Kinase of Streptococcus pneumoniae. Antibiotics.

[21] Mehrbod P., Hudy D., Shyntum D., Markowski J., Los M.J., Ghavami S. (2020). Quercetin as a Natural Therapeutic Candidate for the Treatment of Influenza Virus. Biomolecules.

[22] Wu W., Li R., Li X., He J., Jiang S., Liu S., Yang J. (2015). Quercetin as an Antiviral Agent Inhibits Influenza A Virus (IAV) Entry. Viruses.

[23] Kumar P., Sharma S., Khanna M., Raj H.G. (2003). Effect of Quercetin on lipid peroxidation and changes in lung morphology in experimental influenza virus infection. Int. J. Exp. Pathol..

[24] Raju T.A., Lakshmi A.N., Anand T., Rao L.V., Sharma G. (2000). Protective effects of quercetin during influenza virus-induced oxidative stress. Asia Pac. J. Clin. Nutr..

[25] Schafer M.J., White T.A., Iijima K., Haak A.J., Ligresti G., Atkinson E.J., Oberg A.L., Birch J., Salmonowicz H., Zhu Y. (2017). Cellular senescence mediates fibrotic pulmonary disease. Nat. Commun..

[26] Schafer M.J., Miller J.D., LeBrasseur N.K. (2017). Cellular senescence: Implications for metabolic disease. Mol. Cell Endocrinol..

[27] Xu M., Pirtskhalava T., Farr J.N., Weigand B.M., Palmer A.K., Weivoda M.M., Inman C.L., Ogrodnik M.B., Hachfeld C.M., Fraser D.G. (2018). Senolytics improve physical function and increase lifespan in old age. Nat. Med..

[28] Camell C.D., Yousefzadeh M.J., Zhu Y., Prata L., Huggins M.A., Pierson M., Zhang L., O’Kelly R.D., Pirtskhalava T., Xun P. (2021). Senolytics reduce coronavirus-related mortality in old mice. Science.

[29] Saccon T.D., Nagpal R., Yadav H., Cavalcante M.B., Nunes A.D.C., Schneider A., Gesing A., Hughes B., Yousefzadeh M., Tchkonia T. (2021). Senolytic Combination of Dasatinib and Quercetin Alleviates Intestinal Senescence and Inflammation and Modulates the Gut Microbiome in Aged Mice. J. Gerontol. A Biol. Sci. Med. Sci..

[30] Hickson L.J., Langhi Prata L.G.P., Bobart S.A., Evans T.K., Giorgadze N., Hashmi S.K., Herrmann S.M., Jensen M.D., Jia Q., Jordan K.L. (2019). Senolytics decrease senescent cells in humans: Preliminary report from a clinical trial of Dasatinib plus Quercetin in individuals with diabetic kidney disease. EBioMedicine.

[31] Lorenzo E.C., Torrance B.L., Keilich S.R., Al-Naggar I., Harrison A., Xu M., Bartley J.M., Haynes L. (2022). Senescence-induced changes in CD4 T cell differentiation can be alleviated by treatment with senolytics. Aging Cell.

[32] Larsson N.G., Wang J., Wilhelmsson H., Oldfors A., Rustin P., Lewandoski M., Barsh G.S., Clayton D.A. (1998). Mitochondrial transcription factor A is necessary for mtDNA maintenance and embryogenesis in mice. Nat. Genet..

[33] Falkenberg M., Gaspari M., Rantanen A., Trifunovic A., Larsson N.G., Gustafsson C.M. (2002). Mitochondrial transcription factors B1 and B2 activate transcription of human mtDNA. Nat. Genet..

[34] Shi Y., Dierckx A., Wanrooij P.H., Wanrooij S., Larsson N.G., Wilhelmsson L.M., Falkenberg M., Gustafsson C.M. (2012). Mammalian transcription factor A is a core component of the mitochondrial transcription machinery. Proc. Natl. Acad. Sci. USA.

[35] Lee W.H., Higuchi H., Ikeda S., Macke E.L., Takimoto T., Pattnaik B.R., Liu C., Chu L.F., Siepka S.M., Krentz K.J. (2016). Mouse Tmem135 mutation reveals a mechanism involving mitochondrial dynamics that leads to age-dependent retinal pathologies. Elife.

[36] Lewis S.A., Takimoto T., Mehrvar S., Higuchi H., Doebley A.L., Stokes G., Sheibani N., Ikeda S., Ranji M., Ikeda A. (2018). The effect of Tmem135 overexpression on the mouse heart. PLoS ONE.

[37] Sakabe I., Hu R., Jin L., Clarke R., Kasid U.N. (2015). TMEM33: A new stress-inducible endoplasmic reticulum transmembrane protein and modulator of the unfolded protein response signaling. Breast Cancer Res. Treat..

[38] Kumar R., Mohan N., Upadhyay A.D., Singh A.P., Sahu V., Dwivedi S., Dey A.B., Dey S. (2014). Identification of serum sirtuins as novel noninvasive protein markers for frailty. Aging Cell.

[39] Wu Y., Chen L., Wang Y., Li W., Lin Y., Yu D., Zhang L., Li F., Pan Z. (2015). Overexpression of Sirtuin 6 suppresses cellular senescence and NF-kappaB mediated inflammatory responses in osteoarthritis development. Sci. Rep..

[40] Smogorzewska A., van Steensel B., Bianchi A., Oelmann S., Schaefer M.R., Schnapp G., de Lange T. (2000). Control of human telomere length by TRF1 and TRF2. Mol. Cell Biol..

[41] Li Q., Xiao H., Isobe K. (2002). Histone acetyltransferase activities of cAMP-regulated enhancer-binding protein and p300 in tissues of fetal, young, and old mice. J. Gerontol. A Biol. Sci. Med. Sci..

[42] Golomb L., Sagiv A., Pateras I.S., Maly A., Krizhanovsky V., Gorgoulis V.G., Oren M., Ben-Yehuda A. (2015). Age-associated inflammation connects RAS-induced senescence to stem cell dysfunction and epidermal malignancy. Cell Death Differ..

[43] Capucetti A., Albano F., Bonecchi R. (2020). Multiple Roles for Chemokines in Neutrophil Biology. Front. Immunol..

[44] Tang Y., Fung E., Xu A., Lan H.Y. (2017). C-reactive protein and ageing. Clin. Exp. Pharmacol. Physiol..

[45] Saez P.J., Shoji K.F., Aguirre A., Saez J.C. (2014). Regulation of hemichannels and gap junction channels by cytokines in antigen-presenting cells. Mediat. Inflamm..

[46] Di Virgilio F., Sarti A.C., Coutinho-Silva R. (2020). Purinergic signaling, DAMPs, and inflammation. Am. J. Physiol. Cell Physiol..

[47] Kameritsch P., Renkawitz J. (2020). Principles of Leukocyte Migration Strategies. Trends Cell Biol..

[48] Schallmoser K., Bartmann C., Rohde E., Bork S., Guelly C., Obenauf A.C., Reinisch A., Horn P., Ho A.D., Strunk D. (2010). Replicative senescence-associated gene expression changes in mesenchymal stromal cells are similar under different culture conditions. Haematologica.

[49] Wilbanks A., Zondlo S.C., Murphy K., Mak S., Soler D., Langdon P., Andrew D.P., Wu L., Briskin M. (2001). Expression cloning of the STRL33/BONZO/TYMSTRligand reveals elements of CC, CXC, and CX3C chemokines. J. Immunol..

[50] Varela I., Cadinanos J., Pendas A.M., Gutierrez-Fernandez A., Folgueras A.R., Sanchez L.M., Zhou Z., Rodriguez F.J., Stewart C.L., Vega J.A. (2005). Accelerated ageing in mice deficient in Zmpste24 protease is linked to p53 signalling activation. Nature.

[51] Liu B., Wang J., Chan K.M., Tjia W.M., Deng W., Guan X., Huang J.D., Li K.M., Chau P.Y., Chen D.J. (2005). Genomic instability in laminopathy-based premature aging. Nat. Med..

[52] Hall B.M., Balan V., Gleiberman A.S., Strom E., Krasnov P., Virtuoso L.P., Rydkina E., Vujcic S., Balan K., Gitlin I.I. (2017). p16(Ink4a) and senescence-associated beta-galactosidase can be induced in macrophages as part of a reversible response to physiological stimuli. Aging.

[53] Guth A.M., Janssen W.J., Bosio C.M., Crouch E.C., Henson P.M., Dow S.W. (2009). Lung environment determines unique phenotype of alveolar macrophages. Am. J. Physiol. Lung Cell Mol. Physiol..

[54] Fujihara M., Muroi M., Tanamoto K., Suzuki T., Azuma H., Ikeda H. (2003). Molecular mechanisms of macrophage activation and deactivation by lipopolysaccharide: Roles of the receptor complex. Pharmacol. Ther..

[55] Halbower A.C., Mason R.J., Abman S.H., Tuder R.M. (1994). Agarose infiltration improves morphology of cryostat sections of lung. Lab. Investig..

