# Supplementary material for: Impact of Senolytic Treatment on Gene Expression in Aged Lung
Source: Int J Mol Sci. 2023 Apr 21;24(8):7628. doi: 10.3390/ijms24087628 (PMC10145650; doi:10.3390/ijms24087628)
Supplement: Supplementary file 1 [file ijms-24-07628-s001.zip › ijms-2225789-supplementary.pdf]

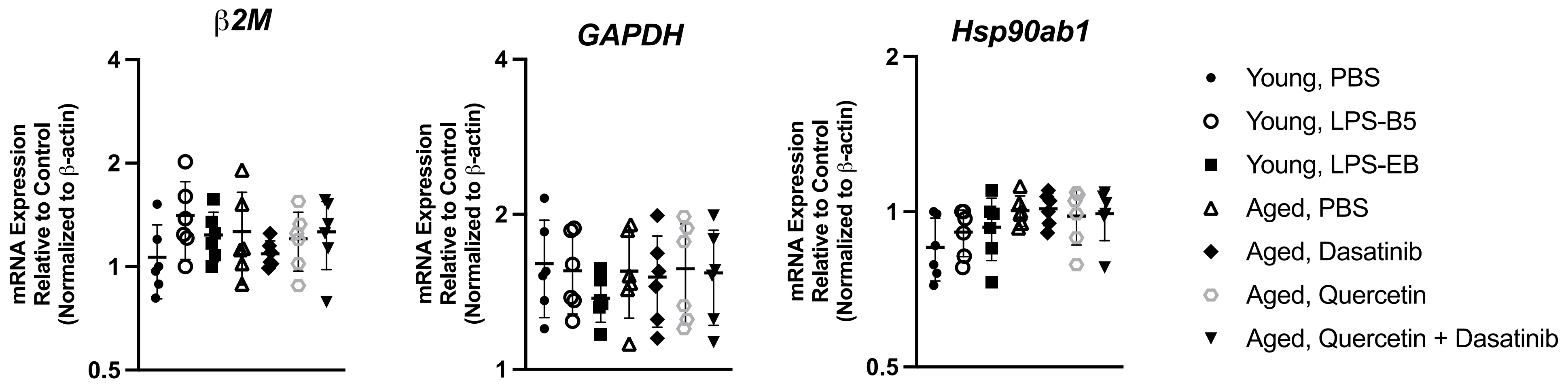


Supplemental Figure S1: Comparison of mRNA expression in lung isolated from control and treated young and aged mice.
